# Animal models of spontaneous activity in the healthy and impaired auditory system

**DOI:** 10.3389/fncir.2015.00019

**Published:** 2015-04-30

**Authors:** Jos J. Eggermont

**Affiliations:** Department of Physiology and Pharmacology, Department of Psychology, University of CalgaryCalgary, AB, Canada

**Keywords:** spontaneous firing rate, neural synchrony, burst firing, auditory nerve, inferior colliculus, auditory cortex, noise trauma, tinnitus

## Abstract

Spontaneous neural activity in the auditory nerve fibers and in auditory cortex in healthy animals is discussed with respect to the question: Is spontaneous activity noise or information carrier? The studies reviewed suggest strongly that spontaneous activity is a carrier of information. Subsequently, I review the numerous findings in the impaired auditory system, particularly with reference to noise trauma and tinnitus. Here the common assumption is that tinnitus reflects increased noise in the auditory system that among others affects temporal processing and interferes with the gap-startle reflex, which is frequently used as a behavioral assay for tinnitus. It is, however, more likely that the increased spontaneous activity in tinnitus, firing rate as well as neural synchrony, carries information that shapes the activity of downstream structures, including non-auditory ones, and leading to the tinnitus percept. The main drivers of that process are bursting and synchronous firing, which facilitates transfer of activity across synapses, and allows formation of auditory objects, such as tinnitus.

## Spontaneous Neural Activity; Noise or Information Carrier?

Spontaneous neural activity is very often considered as neural noise that sets limits on sensory performance. This neural noise idea has been the basis for the optimal processor model in psychoacoustics that typically worked on activity in auditory nerve fibers (Green, 1964; Siebert, 1965) and tried to extract the stimulus-induced activity from the spontaneous noise in these fibers. The concept of internal noise—albeit not limited to spontaneous firing—is fundamental in signal detection theory (Green and Swets, 1966). The alternative, already mentioned in Rodieck et al. (1962), investigating spontaneous firings in the cochlear nucleus, is that spontaneous firing is a carrier of information. The difference in these two hypotheses becomes visible when the effects of stimuli are taken into account. If spontaneous activity acts as noise one expects any stimulus induced activity to be additive to the spontaneous one. In contrast, when the spontaneous activity acts as carrier of information, one expects stimuli to modulate the spontaneous firings, i.e., a multiplicative action. I will compare the normal spontaneous activity in auditory nerve fibers (ANFs) and in primary auditory cortex (AI) with respect to these two hypotheses.

### Auditory Nerve Firings

The neural noise hypothesis for spontaneous activity in auditory nerve fibers was apparently boosted by Kiang et al.’s (1965) extensive studies on ANF activity in the cat, including spontaneous firing properties. The inter-spike interval distribution of spontaneous firings in ANFs strongly suggested an underlying Poisson process with dead time (the refractory period). Geisler et al. (1985) also found that for high spontaneous firing rate (SFR) ANFs in cat the mean of the inter-spike intervals was nearly equal to their standard deviation (SD). Siebert (1965), referring to Kiang et al. (1965), wrote: “Recent electrophysiological studies of the activity in response to acoustic stimuli of single primary afferent neurons in the VIIIth nerve of mammals strongly suggest that the spike activity is inherently stochastic. … Since the only way in which auditory information can reach the more central parts of the nervous system is via the VIIIth nerve, the effective “neural noise” implied by such stochastic “coding” of auditory information must set some sort of limits on auditory discriminations.”

A decade later, Liberman (1978) raised cats in a soundproof room to exclude all potential causes from noise exposure on ANF firing properties. He demonstrated different synaptic noise sources reflected in “units with spontaneous rates greater than 18 spikes/s (sp/s) comprise a distinct and homogeneous group with respect to threshold. The suggestion that the units with rates below 18 sp/s fall into two threshold classes rather than one is most convincing when the overall (hearing) sensitivity of the experimental animals is exceptionally good.” Later on, using intracellular labeling, a relation was found between SFR and ANF diameter in the cat (Liberman, 1982; Liberman and Oliver, 1984). Systematic differences were also found (Merchan-Perez and Liberman, 1996) “in synaptic ultrastructure among fibers of the three SFR groups: with decreasing SFR, the size and complexity of the synaptic body (a presynaptic specialization characteristic of the peripheral afferent synapses in all hair cell systems and some other peripheral receptors) tend to increase, as does the associated number of synaptic vesicles.” These correspondences (see also Jackson and Carney, 2005) suggested that the difference in SFR is functional, making it unlikely that it is just neural noise. Augmenting this was the finding that ANFs with different SFR tended to project to different cell groups in the anteroventral cochlear nucleus (AVCN). Liberman (1991) described it as: “the small cell cap was almost exclusively innervated by low- and medium-SFR fibers, i.e., those with the highest acoustic thresholds. Within anterior AVCN, spherical-cell innervation was seen from all SFR groups, whereas almost all multipolar cell innervation was from low- and medium-SFR fibers. In the posterior AVCN, multipolar-cell innervation was equally likely from all SFR groups, whereas globular cells were preferentially contacted by high-SFR fibers.”

Javel et al. (1988) showed for high SFR ANFs of the cat that the phase-locking (vector strength) of firings to the frequency of a tone showed a nearly 20 dB lower detection threshold than found on the basis of increases in driven firing rate. The latter, however, correlated better with behavioral thresholds. This suggests that the SFR can be modulated even by a sub-threshold stimulus, clearly in contradiction to the notion that SFR in auditory nerve fibers is noise, and supporting an information carrier function.

### Auditory Cortex

#### Spontaneous Firing Rates

Spontaneous activity in cat auditory cortex was initially examined in paralyzed cats (Goldstein et al., 1968). Spontaneous activity was varied with some cells (*n* = 41) having SFR <1 sp/s but in a few others (*n* = 2) >35 sp/s, the remaining 60 cells had SFRs between 1–35 sp/s. Eggermont and colleagues performed studies on spontaneous activity in AI of ketamine-anesthetized cats. In a series of studies (Eggermont, 1992, 1994; Ochi and Eggermont, 1996; Eggermont and Kenmochi, 1998; Kimura and Eggermont, 1999; Valentine and Eggermont, 2001; Noreña and Eggermont, 2003), comprising a total of 2028 units, we found in each study units with SFRs between 0.02 and 30 sp/s, and with a mean between 1.9–3.5 sp/s. I will present these findings in more detail.

Eggermont (1992) recorded at least 15 min of spontaneous activity from each of 312 neurons in 9 adult ketamine-anesthetized cats from all layers of primary auditory cortex, and studied their pair-wise cross-correlation. The findings were described as: “for the 181 single-electrode pairs the percentage of unilateral excitation pairs (42%) was about the same as the percentage of common input pairs (38%), For the 77 unilateral excitation pairs a presynaptic spike produced on average 0.4 postsynaptic spikes, with 61 values <0.5 and only 16 above that value. The values >0.5 were consistently found in cases where the postsynaptic neuron was bursting. For the 297 dual-electrode pairs all but one of the 184 significant correlations were indicative of common input.” In a subsequent study in ketamine-anesthetized cats, Eggermont (1994) investigated the effect of stimulation on the correlation strength. This study clearly indicated that the differences in cross-correlation strength between spontaneous and stimulus-driven activity could not be explained by an additive effect of the stimulus-induced correlations onto those of the spontaneous correlations, as is generally assumed by the “shift-predictor” correction procedure (Perkel et al., 1967). In this procedure, one spike train is shifted by one or more stimulus periods, and the resulting cross-correlogram is then interpreted as stimulus correlation and subtracted from the non-shifted correlogram. In our study, the remaining correlation after correction was typically smaller than the spontaneous cross-correlation. The reason may be that stimulation suppresses the SFR, and this in turn may lead to lower cross-correlation strength (see below; Britvina and Eggermont, 2008). This suggests that in auditory cortex the correlated spontaneous activity cannot be characterized as noise.

#### Spontaneous Burst Firing

**Figure 1:**
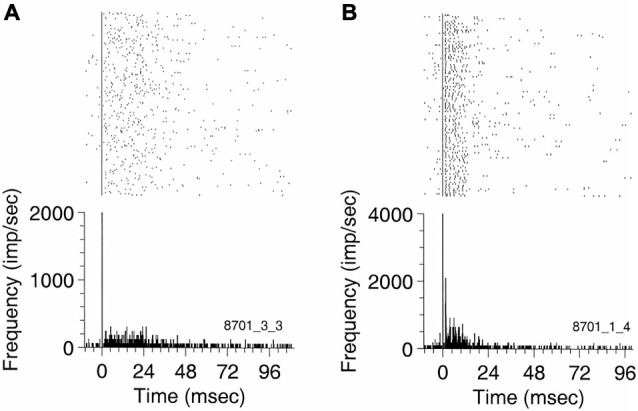
**Peri-event rasters for (A) a non-stereotyped bursting unit recorded in AI, and (B) a stereotyped burster recorded in ANTERIOR AUDITORY FIELD (AAF) simultaneously with the previous unit**. The rasters and the PSTH were triggered on the first spike of the bursts. Ten ms of spontaneous activity are shown before the first burst spike. The burst in **(A)** has a duration of about 50 ms. The AAF unit in **(B)** exhibits a well-defined ISI of 2 ms (bin width 0.5 ms) and has an average duration of about 25 ms. Reprinted from Hearing Research, Vol 154, Pamela A Valentine, Jos J Eggermont, Spontaneous burst-firing in three auditory cortical fields: its relation to local field potentials (LFP) and its effect on inter-area cross-correlations. Pages 146–157, Copyright 2001, with permission from Elsevier.

Eggermont et al. (1993) then described the occurrence of spontaneous burst firing in keteamine-anesthetized cat AI. Bursts with durations less than 50 ms were “characterized by relatively well-defined intervals between the first two spikes (3–15 ms) in the burst followed by intervals with large spread (range 4–50 ms) and increasing modal interval value. The typical five-spike template that described a spontaneous burst in adult cat AI featured spikes at 0, 3.3, 14.6, 27.2, and 34.8 ms, (0 indicating the start of the burst). Bursts with fewer spikes showed larger intervals between the first three spikes.” The probability of occurrence of isolated spikes, pairs, triplets, etc. showed a power-law dependence on SFR with a coefficient that was significantly lower than expected under Poisson firing conditions. A subgroup or neurons with the highest SFRs showed firing behavior close to Poisson and they showed less bursting. Also in the cat, Valentine and Eggermont (2001) found “burst-firing occurred in 85% of 371 units studied, and in 48 (15%) thereof there were at least 100 bursts per 15 min. Neurons in AI were bursting at a significantly higher rate, but with fewer spikes per burst, than units in (secondary auditory cortex) AII”. In addition, we found that burst firing was not synchronized across cortical areas, so that it cannot attributed to a general cortical state characterized by spindling induced by the ketamine anesthesia. Only a few stereotyped bursting neurons were found, notably in anterior auditory field (AAF; Figure 1).

#### Effects of Stimulation on Spontaneous Cortical Activity

Eggermont (2006) recorded spontaneous and stimulus-driven spiking activity from auditory cortex in ketamine-anesthetized cats using multi-electrode arrays. Cross-correlograms were calculated for spikes recorded on separate microelectrodes, and corrected for mean SFR and effects of local field potential (LFP)-spindling activity. A pair-wise cross-correlation matrix was constructed for the peak values of the correlograms. Hierarchical clustering was then performed on the cross-correlation matrix for silence and five stimulus conditions. These neuron clusters reflected the firing synchrony across cortical patches of several mm^2^ in area. The most striking result was that the cluster locations and size were very similar for spontaneous activity and multi-tone-stimulus evoked activity.

**Figure 2:**
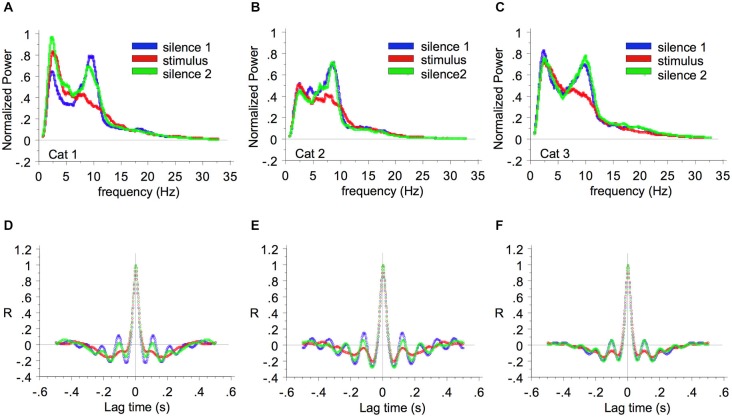
**Suppression of spindle wave power by multi-frequency sound**. The average LFP power spectra are shown for cat 1 **(A)**, cat 2 **(B)**, and cat 3 **(C)**. The power during the first 15-min period of silence, the subsequent 15-min period of multi-frequency stimulation, and the second 15-min period of silence are represented by blue, red, and green lines respectively. Parts **(D)**, **(E)**, and **(F)** show the corresponding auto-correlation functions. Reprinted from Neuroscience, Vol 151, Tatiana Britvina, Jos J Eggermont, Multi-frequency auditory stimulation disrupts spindling activity in anesthetized animals. Pages 888–900, Copyright 2008, with permission from IBRO (2008).

Britvina and Eggermont (2008) found that in ketamine-anesthetized cat AI, multi-frequency tonal stimulation suppressed spontaneous spindle waves, as shown by the decrease of spectral power within the spindle frequency range during stimulation as compared with the previous silent period (Figure 2). They showed that the percentage suppression was independent of the power of the spontaneous spindle waves, and that the suppression of spindle power occurred within one spindle period (≤150 ms) after stimulus onset. The finding that spontaneous spindle oscillations can be modulated by stimuli suggests that this spontaneous LFP activity cannot be considered as “noise”.

In neocortex alternating “DOWN” states with near absence of spontaneous activity and “UP” states of persistent spontaneous firing occur. Luczak et al. (2007) simultaneously recorded populations of 50–200 cortical neurons in layer V of anesthetized (urethane or ketamine) and awake rats. Each neuron displayed a bursting spike pattern during these spontaneous UP states. Spike timing in these bursts was most precise during the first ~100 ms after UP state onset, and became more variable as the UP state continued. In auditory cortex, these bursts had a stereotyped make up, that was similar between UP state onset periods and stimulus onset periods, but the rate and also precise relative timing of spikes varied between stimuli (Luczak et al., 2013), suggesting a modulatory function of stimulation on the bursting pattern. The finding by Chen et al. (2013) that “spontaneously-recurring UP states evoked in these (dendritic) spines “patterned” calcium activity that may control consolidation of synaptic strength following epochs of sensory stimulation” also indicates that spontaneous activity in cortex is not noise.

All in all, these studies suggest that in auditory cortex, just as in auditory nerve fibers, the spontaneous firing activity is not just neural noise but plays a dominant part in information processing.

#### Anesthesia Effects on Spontaneous Firing

Anesthesia will affect SFRs in a way that depends on depth and type of anesthesia. Barbiturates and urethane have a strong effect, ketamine very little. In contrast to studies that measure anesthesia effects on stimulus-evoked activity, there are only a few studies that compared SFRs under anesthesia and awake. Torterolo et al. (2002) reported that pentobarbital anesthesia significantly reduced the SFR in the IC of guinea pigs from (mean ± SEM) 17.8 ± 1.7 sp/s in the awake state to 6.6 ± 0.6 sp/s. These SFRs in the awake state are similar to those measured under ketamine-xylazine anesthesia in the ICC of guinea pigs (mean ± SD): 19.4 ± 19.9 sp/s (Syka et al. (2000). Ter-Mikaelian et al. (2007) recorded from inferior colliculus and auditory cortex (AI) in ketamine anesthetized, and awake gerbils. They found no significant difference in SFR in the awake or anesthetic state in either IC (Mann–Whitney *U* test, one-tailed, *p* = 0.2161; *n* = 94; median, 3.8 sp/s anesthetized and 2.4 awake) or AI (Mann–Whitney *U* test, one-tailed, *p* = 0.1343; *n* = 74; median, 1.6 sp/s anesthetized and 1.0 awake). Huang et al. (2013) compared response properties in rat AI in urethane and ketamine anesthesia. They found that the mean SFR was 2.5 ± 0.6 sp/s under urethane, and 5.6 ± 1.2 sp/s under ketamine/xylazine (*p* = 0.028, Kruskal-Wallis test).

Anesthesia also changes the prevalence and properties of burst firing in neocortex. Ketamine anesthesia results in spindle-like LFP activity in the 5–15 Hz range in cat auditory cortex, depending on the depth of the anesthesia (Britvina and Eggermont, 2008), and is synchronized with bursts of action potentials. Erchova et al. (2002) recording from rat somatosensory cortex during light, intermediate and deep levels of urethane anesthesia. At all levels, spontaneously action potential firing at a single electrode tended to be clustered into “bursts”. With increasing level of anesthesia, burst firing became more prominent (duration increased from 81 ms to 160 ms) and rhythmic. Burst frequency decreased from 1.6 to 0.4 burst/s and fewer spikes occurred outside bursts, leading to a decrease in the overall SFR from 5.9 to 2.8 sp/s.

## Effects of Noise-Induced Hearing Loss

Hearing loss is frequently accompanied by tinnitus. As tinnitus may be a consequence of increased SFR (Eggermont and Roberts, 2004; Roberts et al., 2010) the study of SFR at various locations in the auditory system has met with increasing interest in the last decade. Here I follow part of the narrative from Chapter 7 in “The Neuroscience of Tinnitus” (Eggermont, 2012) while adding more recent findings. The issue I will address: Is increased SFR in tinnitus “noise”?

### Auditory Nerve Fibers

Let us first look at the effects of noise-induced hearing loss (NIHL) on SFRs in auditory nerve fibers. Liberman and Kiang (1978) “exposed cats for 1–4 h to narrow band or broadband noise with levels between 100–117 dB SPL, and recorded ANF activity at 15–305 days after the trauma. Frequency regions with unaffected thresholds typically showed the normal bimodal distribution of SFRs. For units in the hearing loss region, the SFR distribution had lost its normal bimodal appearance. There was a low-SFR region <20 sp/s and a high-SFR region from 20–100 sp/s mostly with SFRs between 10–40 sp/s. Units that had become unresponsive to sound generally showed spontaneous bursting or no spontaneous activity at all. An important finding is that SFRs were hardly ever increased after noise trauma (Liberman and Kiang, 1978).

### The Auditory Brainstem

Superficial multi-unit recordings, likely from fusiform cells, in the dorsal cochlear nucleus (DCN) of hamsters with 10 kHz, 125 dB SPL, 4 h induced noise trauma (Kaltenbach et al., 1998, 2000) showed strongly increased SFR. No correlation between SFR increase and hearing loss was found. In these hamsters, mean SFRs increased from below normal levels at day 2 post-exposure to higher than normal levels at day 5. The mean SFR continued to increase gradually over the next 6 months. Hamsters exposed to a lower level sound (10 kHz, 80 dB SPL, 4 h), showed multi-unit SFRs in the DCN that were already increased above control levels at 1 h post-exposure and significantly increased at 2 days after exposure (Kaltenbach et al., 2005). Recording from single units of the DCN in hamsters, Finlayson and Kaltenbach (2009) showed average SFRs of 8.7 sp/s in controls and 15.9 sp/s after exposure to a 10 kHz tone at a level of 115 dB SPL for 4 h. The highest increases in SFR were found in the fusiform cell layer. Approximately half of the increase in SFR in exposed animals was accounted for by an increase in bursting activity. This effect may only be transient; SFRs were significantly higher than normal at 1 week following noise damage, whereas at 2 weeks post-noise damage SFRs were no longer significantly different from control (Shore and Zhou, 2006). This may not be in agreement with the long lasting effects in superficial recordings shown by Kaltenbach et al. (2000), which were also attributed to fusiform cells. Removing spontaneous input to the DCN in hamsters by cochlear ablation 30 days after the exposure had no significant effect on SFRs, suggesting that the increased SFRs at that time were intrinsically generated (Zacharek et al., 2002). This is consistent with findings by Koerber et al. (1966) showing that SFR in DCN of normal hearing cats did not change after cochlear ablation.

Ma and Young (2006) exposed cats to a 250 Hz band of noise centered at 10 kHz that was presented at 105–120 dB SPL for 4 h. After a one-month recovery period, neural activity was recorded in the DCN of a decerebrated preparation, which eliminates corticofugal activity towards the DCN among other effects. The threshold shift, determined from CAP audiograms, showed a sharp threshold elevation of about 60 dB for neurons with CFs above the 5–10 kHz lower-edge frequency of the hearing loss. In contrast to the above-described results in hamsters that were subjected to a similar exposure level and duration, SFRs in fusiform cells with elevated thresholds were not increased over those in unexposed animals. This could suggest a species difference as the recovery period is in the range where increased SFRs were seen in hamsters. As I remarked earlier (Eggermont, 2012): “The different delays between the exposure and the recording may have had an effect as well; Shore and Zhou (2006) showed that there was only a transient elevation for 1–2 weeks after the trauma. Finally, the recovery of the cats could have been in a noisy acoustic environment, which may have prevented the increase in SFR (see Noreña and Eggermont, 2005, 2006).”

Vogler et al. (2011) exposed guinea pigs for 2 h to a 10 kHz tone presented at 124 dB SPL. After a 2-week recovery period, the mean SFR in the VCN (VCN) of noise-exposed ears (*N* = 189) was significantly elevated (about a factor two) compared to sham controls (*N* = 143). This was more evident in primary-like and onset types of neurons. In addition, mechanical damage to the high frequency region of the cochlea (*N* = 258) showed similar results as noise exposure, suggesting that it is the hearing loss and not the induction method that causes the SFR changes.

### The Inferior Colliculus

Ma et al. (2006) exposed CBA/J mice for 1 h to a 0.5 oct. band of noise centered around 16 kHz, 103 dB SPL. Bilaterally exposed mice fairly shortly after the exposure showed increases in the SFR of neurons in the central nucleus of the inferior colliculus (ICC) with tuning near the exposure frequency. However, the median SFR (6.0 sp/s) was not significantly different from controls, who had SFRs between 0 and 30 sp/s, with a median SFR = 4.1. No changes in burst-firing activity in the ICC were found for bilateral exposed mice. However, they reported changes in temporal aspects of firing in the protected ear after unilateral exposure. The contralateral ICC showed increased median ISIs, reduced SFR, and a significant increase in burst firing (Ma et al., 2006).

Vogler et al. (2014), using the same exposure as in the VCN (Vogler et al., 2011), also showed increased SFR in the ICC in regions corresponding to the frequencies at which there was peripheral hearing loss (12–20 kHz). Most unit types, with the exception of onset cells, showed a significantly increased mean SFR. Thus, in contrast to findings in the VCN, hyperactivity in the ICC was not confined to a particular cell type. This was confirmed by Ropp et al. (2014) in Sprague-Dawley rats who showed in ICC a median pre-trauma SFR = 10.4 sp/s and a post-trauma one of 14.1 sp/s. They found that abnormal SFRs were restricted to target neurons of the VCN. So one wonders what increased SFR in the DCN has to do with tinnitus. Nearly identical patterns of hyperactivity were observed in the contralateral and ipsilateral ICC. The elevation in SFR was found for frequencies well below and above the region of maximum hearing loss.

As in the DCN, acoustic trauma (10 kHz tone at 124 dB SPL for 1 h) in guinea pigs did not immediately resut in SFR changes in the ICC. Two weeks recovery after acoustic trauma resulted in more neurons with high SFR compared to control animals, and a significant increase in the average SFR from control (mean = 1.2 sp/s) values (Mulders and Robertson, 2009). Surprisingly, subsequent cochlear ablation, cochlear cooling or kainic acid infusion in the cochlea, resulted in statistically significant decreases in the average SFR in ICC, from 4.5 to 1.4 sp/s in the animals recorded 1 week post exposure and from 7.5 sp/s in the animals that were recorded from more than 4 weeks after the exposure. Thus, at all recovery times (up to 4 weeks) after the exposure, the increased SFR disappeared when cochlear input to the ICC was destroyed. These data suggested that the hyperactivity in the ICC after acoustic trauma was dependent on activity in the contralateral cochlea. The findings also suggest that the ICC SFR is not dependent on the activity in the DCN, which is not affected by ablation 30 days after induction of the noise trauma (Zacharek et al., 2002).

Corroborating evidence (Mulders et al., 2010) came from electrically stimulating the olivocochlear bundle in noise-exposed animals, which is know to decrease ANF activity, and this also resulted in a decrease of the exposure-enhanced SFR in the ICC. There is a transition period between an amplification of peripheral input to intrinsic generation of SFRs in the ICC. Robertson et al. (2013) observed that a “spontaneous afferent drive from the cochlea itself is necessary for the maintenance of hyperactivity up to about 8 weeks post cochlear trauma. After 8 weeks however, ICC hyperactivity becomes less dependent on cochlear input, suggesting that central neurons transition from a state of hyperexcitability to a state in which they generate their own endogenous firing”.

Except one study (Ma et al., 2006), none of the midbrain studies reported on temporal aspects of spontaneous firing.

### Thalamus and Cortex

#### Immediately Post-Trauma

**Figure 3:**
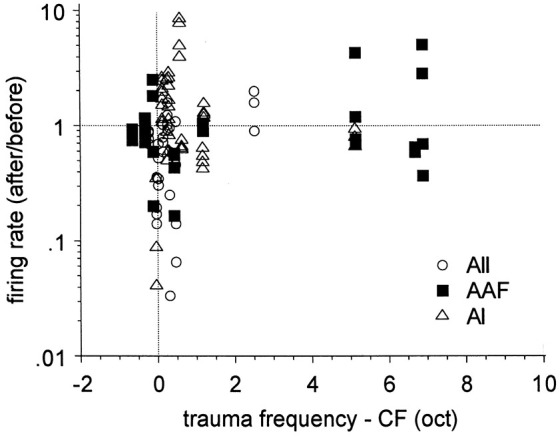
**Effects of pure-tone trauma on single-unit spontaneous firing rates (SFR)**. Shown is the ratio of the firing rates after and before the trauma as a function of the separation (in octaves) of CF and trauma tone frequency (TTF). Reprinted from Hearing Research, Vol 154, Makiko Kimura, Jos J Eggermont, Effects of acute pure tone induced hearing loss on response properties in three auditory cortical fields in cat. Pages 146–162, Copyright 1999, with permission from Elsevier.

Kimura and Eggermont (1999) recorded simultaneously from units in primary auditory cortex, AAF and secondary auditory area of ketamine-anesthetized cats before and immediately after a 30 min exposure to a 93–123 dB SPL pure tone. The frequency of the trauma tone was set 0.5 octave above the highest CF found for the three simultaneous recordings, to investigate effects of diminished lateral inhibition from neurons tuned to frequencies >0.5 oct. above the CFs of the recorded neurons (Figure 3). SFRs increased significantly in AI (from 0.54 to 1.08 sp/s), did not change in AAF (from 0.98 to 0.84 sp/s), and decreased significantly in AII (from 1.22 to 0.76 sp/s). The changes in spontaneous activity as a result of the pure-tone trauma stabilized within a few min after the trauma and for at least up to 30 min.

**Figure 4:**
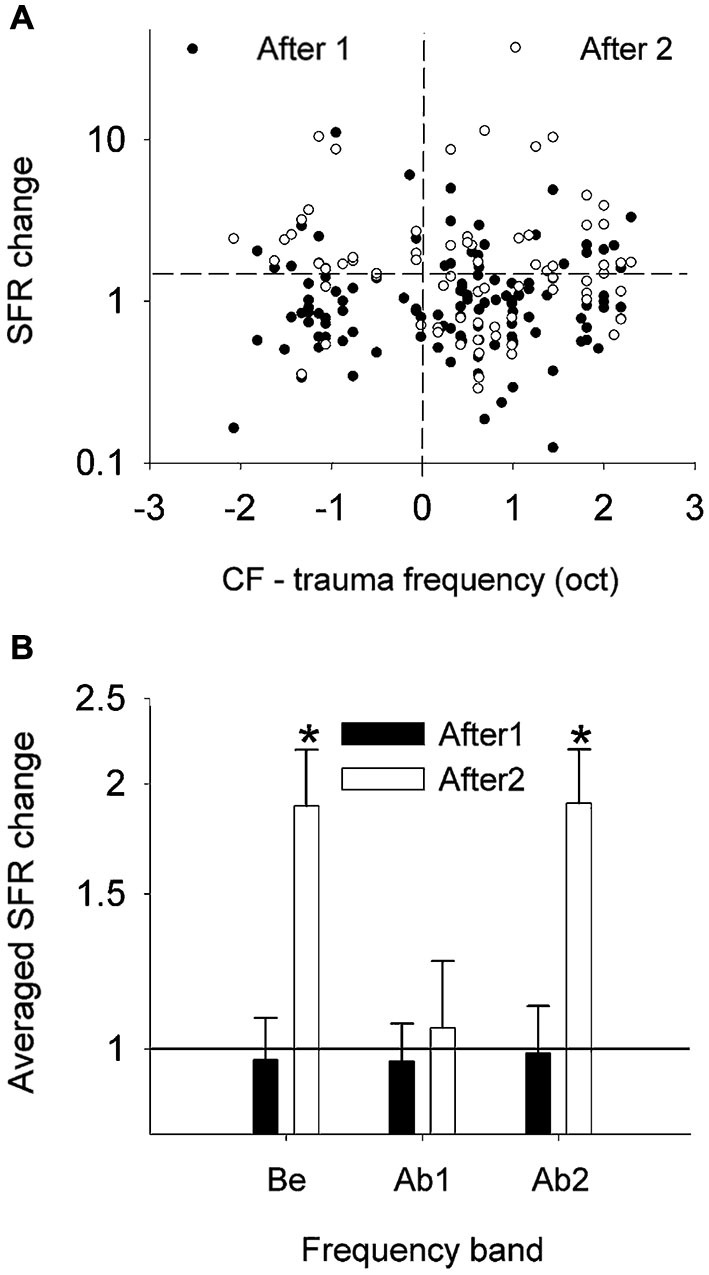
**Effects of the acoustic trauma on the SFR. (A)** Changes in SFR (as a ratio of post/pre) immediately (filled circles) and a few hours (open circles) after the acoustic trauma as a function of the difference in pre-trauma CF and the TF. **(B)** SFR changes averaged (geometric mean) into three frequency bands (±S.E.M., *P < 0.05). Be, CF below the TTF. Ab1, CF-TTF ≤1 octave. Ab2, CF-TTF >1 octave. Reprinted from Hearing Research, Vol 183, A.J. Noreña, J.J. Eggermont, Changes in spontaneous neural activity immediately after an acoustic trauma: implications for neural correlates of tinnitus. Pages 137–153, Copyright 2003, with permission from Elsevier.

Changes in the neural activity in cat AI occurring within a few hours after a 1-h exposure to a 120-dB SPL pure tone (5 or 6 kHz) were further assessed by recording, with two 8-microelectrode arrays, from the same sorted-unit clusters before and after the trauma (Noreña and Eggermont, 2003; Noreña et al., 2003). Immediately after the exposure, the SFR was not significantly changed (Figure 4). Significant increases in SFR did occur after at least 2 h post trauma.

**Figure 5:**
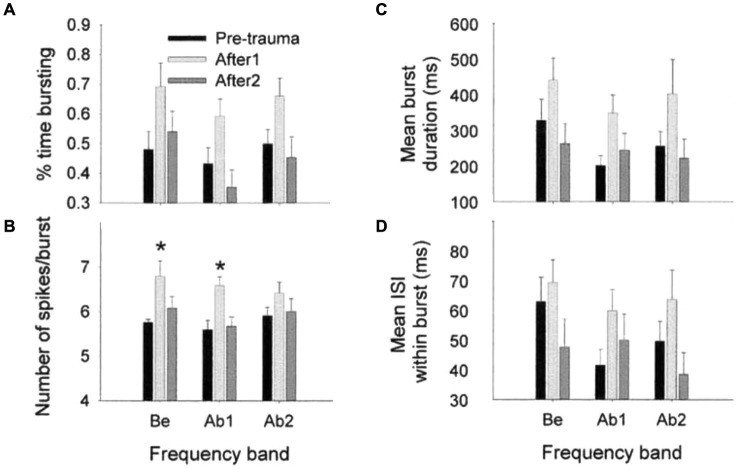
**Effects of an acoustic trauma on burst-firing properties**. The proportion of time of burst-firing **(A)**, the number of spikes by burst **(B)**, the mean burst duration **(C)**, and the mean ISI within a burst **(D)** are shown as a function of the frequency band, in the pre-trauma, After1 and After2 conditions (±S.E.M., *P < 0.01). Be, CF below the TTF. Ab1, CF-TTF ≤1 octave. Ab2, CF-TTF >1 octave. Reprinted from Hearing Research, Vol 183, A.J. Noreña, J.J. Eggermont, Changes in spontaneous neural activity immediately after an acoustic trauma: implications for neural correlates of tinnitus. Pages 137–153, Copyright 2003, with permission from Elsevier.

Spontaneous burst firing in AI was affected by this noise exposure (Noreña and Eggermont, 2003). In total, 497 SU spike trains were analyzed; the Poisson-surprise method (Legéndy and Salcman, 1985), at a surprise value >10 detected bursting activity in 468 of them (94%). Figure 5 illustrates the averaged data for the percentage of time of burst firing (A), the number of spikes per burst (B), the mean burst duration (C) and the mean ISI within a burst (D), at pre- and post-trauma conditions as a function of the frequency band. One observes that the trauma induced an immediate and transitory change in burst-firing properties in the three frequency bands (Be, below the trauma tone frequency (TTF); Ab1, within one oct. above the TTF; Ab2, >1 oct. above the TTF). There was only a significant change in the number of spikes per burst, which was increased immediately after the trauma in Be (*P* < 0.01) and Ab1 (*P* < 0.001) groups. However, when the three frequency bands were combined, unpaired *t*-tests revealed a significant increase in the percentage of time of burst-firing (*P* < 0.0001), number of spikes per burst (*P* < 0.0001), mean burst duration (*P* < 0.0001) and mean ISI within a burst (*P* ≤ 0.001), immediately after the trauma (“After1” condition). The burst properties returned to normal a few hours after the trauma (“After2” condition). However, as we have seen SFRs increased significantly (Figure 4) in the After2 condition. Thus, burst firing and SFRs are not correlated, in fact are negatively correlated.

**Figure 6:**
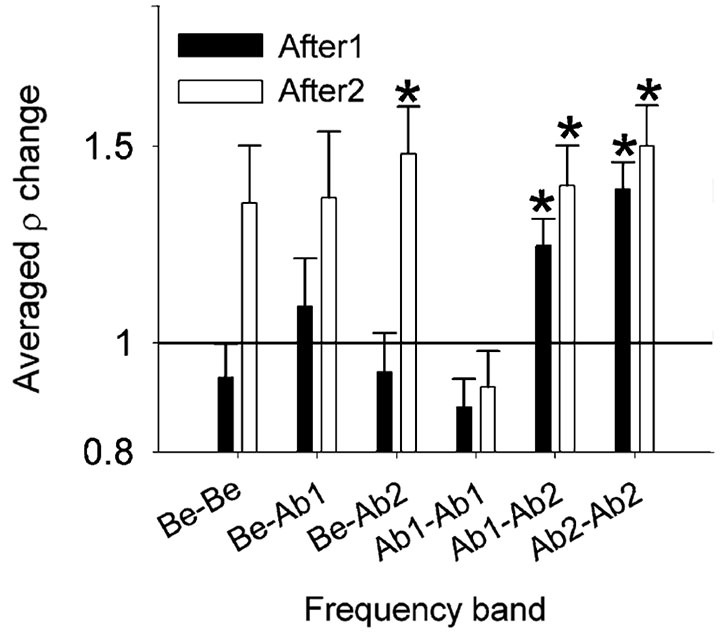
**Effect of the acoustic trauma on the cross-correlation coefficient ρ**. Changes in ρ averaged (geometric mean) into six frequency bands, immediately (After1) and a few hours (After2) after the acoustic trauma (±S.E.M., *P < 0.0083). Immediately after the acoustic trauma (black bars), one notes that ρ is significantly increased in the Ab2- Ab2 group. Be, CF below the TTF. Ab1, CF-TTF ≤1 octave. Ab2, CF-TTF >1 octave. Reprinted from Hearing Research, Vol 183, A.J. Noreña, J.J. Eggermont, Changes in spontaneous neural activity immediately after an acoustic trauma: implications for neural correlates of tinnitus. Pages 137–153, Copyright 2003, with permission from Elsevier.

Spontaneous neural synchrony between spike firing of two neurons is also affected by noise exposure (Noreña and Eggermont, 2003). Figure 6 shows the ratio between post- and pre-trauma cross-correlation coefficient (ρ) averaged (geometric mean) into six groups. One observes that ρ is increased *immediately* after the tone exposure by a factor of about 1.25 for Ab1-Ab2 (*P* = 0.0008) and 1.4 for Ab2-Ab2 groups (*P* < 0.0001). For the After2 condition (well after the trauma), one observes that ρ is significantly increased for the Be-Ab2 (*P = 0.001)*, Ab1-Ab2 (*P* = 0.0004) and Ab2-Ab2 groups (*P* < 0.0001). Again, SFRs increased significantly in the After2 condition over a wider frequency range compared to the changes in ρ.

#### Chronic Changes Post-Trauma

Kalappa et al. (2014) exposed rats unilaterally for 1 h to a 1 oct. band of noise centered on 16 kHz, and with a peak level of 116 dB SPL. Behavior and electrophysiology testing was done 2 months following exposure. At that time there was no remaining hearing loss. Single units in the medial geniculate body (MGB) in awake animals with behavioral (gap-startle test) signs of tinnitus showed significantly enhanced SFR from 4.7 sp/s in controls to 9.1 sp/s after noise exposure. In the gap-startle test, where a gap in a broad-band or narrow-band noise functions as a pre-pulse inhibitor for a startle inducing sound, tinnitus is assumed to fill the gap and (partly) abolishes the inhibitory function of the gap on the startle response (Turner et al., 2006). Burst firing showed a significant increase after noise exposure in: (1) the mean number of bursts per minute (9.2 in control vs. 37.1 in exposed); (2) mean number of spikes in a burst (5.1 in control vs. 6.1 exposed); and (3) mean burst duration (60 ms in control vs. 90 ms after noise exposure). These elevated patterns of neuronal activity and altered bursting showed a significant positive correlation with animals’ scores on the gap-startle test.

Komiya and Eggermont (2000) exposed kittens to a 126 dB SPL tone of 6 kHz for 1 h at both 5 and 6 weeks of age, and recorded from primary auditory cortex 7–16 weeks after the exposure. Single-unit SFRs were significantly higher in reorganized tonotopic map regions (mean 2.3 sp/s) than in unaffected regions (mean 1.4 sp/s). For the littermate controls the mean SFR was 1.3 sp/s and was not CF dependent. Burst firing was not significantly affected by the exposure. Seki and Eggermont (2003) again found increased single-unit SFRs in reorganized tonotopic map regions (mean 3.2 sp/s) compared to the neurons in the non-reorganized map regions (mean 1.8 sp/s) in the same animals and in controls (mean 1.9 sp/s). In these reorganized map regions the peak cross-correlation coefficients were also increased relative to those for unit pairs in the non-reorganized parts. Using the same exposure paradigm, Noreña and Eggermont (2005, 2006) showed again that NIHL and recovery in quiet induces reorganization of the tonotopic map in cat AI. Here the frequencies above 10–15 kHz were no longer represented. In addition the exposure increased the multi-unit SFR (~7 sp/s and a factor 2 larger than in control cats) and neural synchrony (by a factor 1.2) in the reorganized part of AI.

Engineer et al. (2011) induced noise trauma by exposing rats to 1 h of 115-dB SPL, octave-band noise centered at 16 kHz. This resulted in about 15–20 dB permanent hearing loss at 11 weeks post trauma in the frequency region between 4 and 32 kHz. At that time, the tonotopic map was reorganized, and the average multi-unit SFR was significantly increased from 14.3 sp/s to 17.7 sp/s. The degree of synchronization between spontaneous multiunit firings recorded at nearby sites was significantly increased as well.

## Role and Function of Spontaneous Activity

### Changes in the Role of SFR from Auditory Nerve to Cortex

Does the fact that spontaneous inter-spike-intervals in ANFs have a Poisson-like (mean interval equals its variance) structure, whereas in auditory cortex there is a hyperexponential distribution (Eggermont, 1990; Eggermont et al., 1993) with a variance about twice the mean (at least in primary visual cortex; Vogels et al., 1989), indicate a different role of spontaneous activity along the auditory pathway? To evaluate this we will also address whether increased SFR in tinnitus functions as “noise” as implied by the rationale for the gap-startle test (Turner et al., 2006).

First of all, we have to consider if is the designation of spontaneous activity as information carrier or as neural noise is dependent on the level of SFR. In auditory nerve fibers, high SFRs are strongly correlated with low thresholds, i.e., with increased sensitivity. For these units, a low-frequency pure tone can modulate the SFR especially at low sound levels as reflected in the phase-locking of the firings to the tone-period (Javel et al., 1988). This indicates a multiplicative action of the stimulus, suggesting that the spontaneous activity in an information carrier. However, at higher stimulus levels spikes are added to the spontaneous activity. A multiplicative action is hardly possible for the high-threshold, low-SFR ANFs and here stimulation dominantly adds spikes. Considering low SFR as noise, however, is stretching the definition of noise. It is also known that these low-SFR ANFs do not contribute to the CAP even at high stimulus levels (Bourien et al., 2014) and do not affect CAP threshold. These low-SFR units have also been described as vulnerable to noise exposures producing temporary threshold shifts (TTS), which after some delay result in high-threshold ANF loss (Kujawa and Liberman, 2009). This has been suggested (Hickox and Liberman, 2014) to result in increased central gain and through this modulation results in steeper rate-intensity functions and increased SFR. However, they also stated that: “Gap PPI tests” often used to assess tinnitus, revealed limited gap detection deficits in mice with cochlear neuropathy only for certain gap-startle latencies, inconsistent with the presence of tinnitus “filling in the gap.”

Litvak et al. (2003) suggested: “Spontaneous activity helps to faithfully encode stimulus waveforms in the temporal discharge patterns of sensory neurons by allowing these waveforms to be represented by small modulations of ongoing activity. Such modulation coding lowers threshold and mitigates the distortions caused by refractoriness in single neurons. Spontaneous activity may also desynchronize stimulus-driven activity across neurons in a population, thereby allowing a volley principle to operate when the stimulus period is shorter than the neural refractory period. In this view, noise resulting from random spontaneous activity is the price paid for the lower thresholds neural population.”

**Figure 7:**
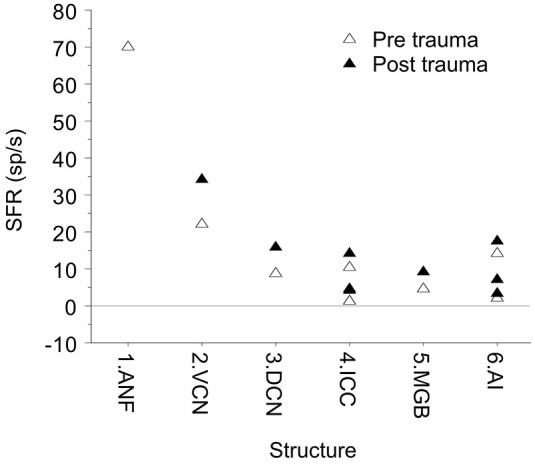
**Mean SFR vs. location in the auditory system**. The data shown are from hamster DCN 8.7 sp/s (Finlayson and Kaltenbach, 2009). From barbiturate guinea pig VCN, 22 sp/s (Vogler et al., 2011). From barbiturate guinea pig ICC, 1.2 sp/s (Mulders and Robertson, 2009) and 1.4 sp/s (Vogler et al., 2014), and from ketamine rat ICC, 10.4 sp/s (Ropp et al., 2014). In medial geniculate body (MGB) of awake Sprague-Dawley rats the SFR was 4.7 sp/s (Kalappa et al., 2014). In auditory cortex, SFRs ranged from 1.9 sp/s (Seki and Eggermont, 2003), to 3.5 sp/s (Noreña and Eggermont, 2003) in ketamine anesthetized cat AI. In pentobarbital anesthetized Sprague-Dawley rats they were surprisingly high at 14.3 sp/s, potentially resulting from multi-unit activity (Engineer et al., 2011).

Is the SFR dependent on the recording site along the auditory pathway? In controls the SFR decreases from ANF (only the mean of high SFRs shown) to in particular the ICC and MGB and then increases somewhat in some auditory cortex recordings, likely because they reflect multi-unit activity in contrast to single-unit activity at more peripheral levels (Figure 7). Could one thus hypothesize that the information carrier function of spontaneous activity be limited to the in general high-SFR in auditory nerve and brainstem and functions only as noise in ICC, MGB and auditory cortex? That also affects how one interprets the recent finding of Buran et al. (2014) that “auditory cortex spontaneous discharge rate can be modulated transiently during task performance, thereby increasing the signal-to-noise ratio and enhancing signal detection.” Clearly, stimuli or tasks modulate the spontaneous activity suggesting the information carrier model, but it also increases the signal-to-noise ratio implying that the spontaneous activity is basically noise.

### The Role of Bursting and Neural Synchrony

After noise trauma, it is often observed that the increase in SFR (Figure 7, full symbols) is accompanied by increased burst firing and increased neural synchrony. Burst firing is uncommon in normal ANFs but is found after noise trauma (Liberman and Kiang, 1978), and also in the DCN (Finlayson and Kaltenbach, 2009) and in the MGB (Kalappa et al., 2014). Ma et al. (2006) did not find any changes in burst-firing activity in the ICC. In AI, changes in burst firing were only transient (and only reflected in the number of spikes in a burst; Noreña and Eggermont, 2003). Bursting was positively correlated with SFR in DCN (after PTS) and MGB (after TTS) and negatively in ANF as it occurred only when the thresholds were very elevated. Noise induced hearing loss very often results in tinnitus as well. This raises the question if increased SFR and burst firing have a causal role in tinnitus generation, which requires at least that increased burst firing is correlated with tinnitus. Only in the DCN and MGB was burst firing correlated with behavioral signs of tinnitus.

These two distinct views of spontaneous activity, either as unwanted noise or as information carrier, may thus determine how one views the neural mechanisms of tinnitus. If one considers spontaneous activity in the auditory system as unwanted noise, the favored concept about tinnitus is likely that it results from too much noise. The suggested neural substrate will then be increased SFR in the auditory system. On the other hand, if one considers spontaneous activity as the information carrier of the brain, sound modulates this firing rate and reorganizes it. In this model external sound can suppress tinnitus. Tinnitus in this model results from increased neural synchrony, i.e., the pathology also reorganizes the spontaneous firing times either in the form of serial correlations, i.e., burst firing, or as parallel correlations, i.e., as synchronous firing among neurons (Eggermont and Tass, 2015).

Both serial and parallel synchrony will enable efficient synaptic transmission and may amplify each other. Eggermont et al. (1993) phrased it as: “Burst firing in neocortex tends to be a communal event; when a neuron is firing in bursts there is a large probability that another adjacent neuron is also firing in bursts (Noda and Adey, 1970). Bursts occurrences of two or more simultaneously recorded neurons often appeared to be temporally close, especially between pairs of neurons recorded by the same electrode (Legéndy and Salcman, 1985). … In a structure such as the neocortex where the connection strengths between pyramidal cells is on average only 0.05 (Abeles, 1982; Eggermont, 1992) burst firing could act as an amplification mechanism of neural activity that could ensure faithful transmission across a synapse. … The timing intervals of increased firing rate for neighboring neurons will overlap to an extent determined by burst duration, and during this interval there will be a tendency to synchronize the events. Co-occurrences of bursts in neurons could therefore recruit those neurons into functional assemblies in a way analogous to the modification of excitatory transmission postulated by Hebb (Neven and Aertsen, 1992).” Both types of enhanced synchrony then may form “objects” of the increased SFRs, which depending on top-down modulating factors, may be experienced as “tinnitus”. This again implies that spontaneous activity is an information carrier.

## Conflict of Interest Statement

The author declares that the research was conducted in the absence of any commercial or financial relationships that could be construed as a potential conflict of interest.
